# Association between maternal HIV infection and birthweight in a tertiary hospital in southern Ethiopia: retrospective cohort study

**DOI:** 10.1186/s13052-020-00834-3

**Published:** 2020-05-24

**Authors:** Andualem Zenebe, Betelhem Eshetu, Samson Gebremedhin

**Affiliations:** 1 Hawassa College of Health Sciences, Hawassa, Ethiopia; 2 School of Public Health, Hawassa University, Hawassa, Ethiopia; 3 School of Public Health, Addis Ababa University, Addis ababa, Ethiopia

**Keywords:** Low birth weight, Maternal HIV infection, Ethiopia

## Abstract

**Background:**

Human Immunodeficiency Virus (HIV) infection and low birth weight (LBW) continue to be significant public health concerns in many low-income countries including Ethiopia. Yet the effect of maternal HIV infection on birth weight has not been thoroughly explored and the existing studies reported opposing findings. We examined the association between maternal HIV infection and LBW in a tertiary hospital in Southern Ethiopia.

**Methods:**

A retrospective cohort study was conducted based on the medical records of 277 HIV-negative and 252 HIV-positive mothers who gave singleton live birth between September 2014 to August 2017 in Hawassa University Comprehensive Specialized Hospital, Southern Ethiopia. The recodes were identified using systematic sampling approach and relevant information were extracted by using pretested extraction form. Multivariable binary logit model was fitted to examine the relationship between the exposure and outcome while adjusting for potential confounders. Adjusted odds ratio (AOR) and 95% confidence intervals (CI) is used for summarizing the findings of the analysis.

**Results:**

The mean (± standard deviation) birth weight of infants born to HIV-negative women (3.1 ± 0.7 kg) was significantly higher than those born to HIV-positive counterparts (3.0 ± 0.6 kg) (*p* = 0.020). The prevalence of LBW was also significantly higher in the HIV-exposed group (22.2%) than the non-exposed group (13.7%) (*p* = 0.011). In the logit model adjusted for multiple covariates, HIV-positive women had four times increased odds than HIV-negative women to give birth to LBW infant(AOR = 4.03, 95% CI: 2.01–8.06). Other significant predictors of LBW were rural place of residence (AOR = 2.04, 95% CI: 1.16–3.60), prenatal anemia (AOR = 3.17, 95% CI: 1.71–5.90), chronic hypertension (AOR = 3.68, 95% CI: 1.10–12.46) and preeclampsia (AOR = 6.80, 95% CI: 3.00–15.38).

**Conclusion:**

Maternal HIV infection is associated with increased odds of LBW. HIV prevention activities are also likely to contribute for the reduction of LBW.

## Background

Low birth weight (LBW) defined as weight at birth less than 2500 g, is the consequence of either preterm birth or intrauterine growth restriction [1]. Globally 21 million births, equivalent to 15% of all births, are born with LBW [2]. The disparity in the burden of LBW between low- and high-income countries is noticeable as the prevalence in the Europe and North America remains below 8% while in sub-Saharan Africa and South Asia it exceeds 14 and 25%, respectively [2]. The World Health Assembly target for a 30% reduction of LBW between 2012 and 2025 remains largely unattainable [3, 4].

In Ethiopia no reliable current data is available on the national prevalence of LBW. In 2004, the United Nations Children’s Fund estimated 20% national prevalence [1]. According to the Ethiopian Demographic and Health Survey (DHS) - 2016, based on the subjective reports of mothers, the prevalence of small size births was estimated at 13% [5]. A recent systematic review classified Ethiopia among the top-10 countries with the highest burden of preterm births [6]. Another systematic review which pooled small-scale studies conducted in different localities of Ethiopia in the last 30 years estimated aggregate prevalence of 17.3% [7].

Low birth weight is proxy indicator of poor maternal-fetal nutrition and leads to multiple short and long-term sequels. Infants born with weight less than 2.5 kg are approximately 20 times more likely to die during infancy than their counterparts [1]. Furthermore, very low birth weight infants tend to have weaker immunity and long-term growth, cognitive and neurologic deficits [8, 9]. LBW is also linked with increased propensity of developing chronic diseases including coronary heart disease and diabetes mellitus later in the adulthood life [10, 11]. LBW imposes huge economic burden on the family, health system, and society [12].

HIV/AIDS remains a major global public health challenge, especially in the sub-Saharan African region. In 2017, nearly 37 million people were living with the virus and an estimated 1.8 million new cases had been reported worldwide [13].With national adult prevalence of 1.1% and more than 600,000 people living with HIV, Ethiopia is one of the most severely HIV-affected countries in the region [5, 14]. In 2011, the prevalence among women in the reproductive age group was 1.9% [15]. According to an estimate, antiretroviral treatment coverage among pregnant women for prevention of mother-to-child transmission (PMTCT) of HIV stood at 70% [16].

The association between maternal HIV-infection and LBW has not been adequately explored and the available studies suggested inconsistent findings [17]. Among others, studies conducted in Malawi [18], Kenya [19], United States of America [20] and Ethiopia [21, 22] witnessed significant association between maternal HIV infection and LBW. Conversely, studies from Nigeria [23], Tanzania [24], India [25] and Italy [26] found no significant birth weight differences between infants born to HIV-positive and -negative women. A systematic review found significant association between maternal HIV infection and LBW; however, the analysis came across with considerable heterogeneity among the original studies, suggesting the relationship is likely to vary across settings [17]. In the current study we examined the association between HIV and LBW status in HU-CSH, Southern Ethiopia.

## Methods

### Study setting

HU-CSH is a university-affiliated tertiary public hospital found in Hawassa city, the capital of Southern Nations Nationalists and People’s Region, Ethiopia. The 400-bed capacity hospital annually provides service for approximately 13,000 inpatient, 95,000 outpatients and serves as a referral facility for more than 10 million population. HU-CSH was purposely selected for the study based on the high case load of HIV-positive women on PMTCT follow-up. In the hospital, in line with the national protocol, provider-initiated counseling and HIV testing is routinely offered to all pregnant women on antenatal care.

### Study design and sampling approach

This retrospective cohort study was conducted in the hospital from January to March, 2018. Births to HIV-positive women were considered as the exposed group; whereas, those born to HIV-negative women constituted the non-exposed group. Ultimately, birthweight of infants born to the two groups were statistically compared with adjustment for selected confounders. All HIV-positive and -negative women who gave singleton live births at HU-CSH from September 2014 to August 2017 were considered eligible for the study, and those with missing medical information about the exposure or outcome status were excluded.

We originally intended to sample the medical records of 568 mother-baby dyads, comprising 284 HIV-positive mothers and 284 HIV-negative mothers. This sample size was reached at using two population proportions formula with specifications of 80% power, 95% confidence level, one-to-one ratio between exposed and non-exposed subjects, and 3.2 and 9.8% expected prevalence of LBW among non-exposed and exposed subjects, respectively [27].

**Fig. 1:**
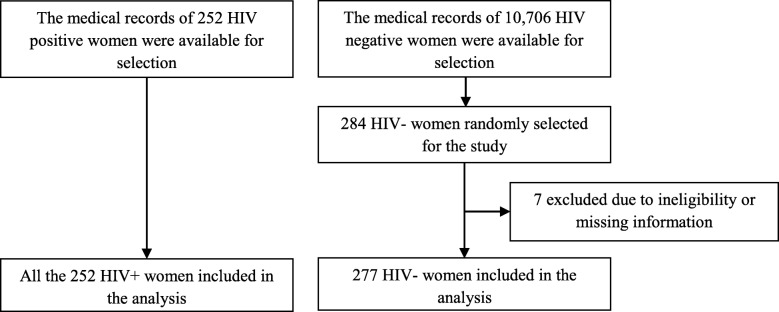
Flow chart of the study

Pertaining to the sampling procedure, initially we identified the list of all HIV-positive and -negative women who gave birth in the facility in the reference period and distinct sampling frames were developed for the two groups. From the available 10,706 HIV-negative women, we selected 284 using systematic random sampling method. However, we included all the available medical records of the HIV-positive women (*n* = 252) because the available numbers were lower than what we initially intended to sample. Ultimately, the selected medical records were accessed and eligibility and completeness of the records were evaluated further (Fig. 1).

### Data collection procedures and study variables

Eight trained supervisors and data collectors extracted the data from the medical records using a structured and pretested data extraction form. For the HIV-negative mothers, information including basic socio-demographic characteristics, birth weight, obstetric history, occurrence of obstetric complications and length of gestation were extracted from ANC or birth records. For HIV-positive mothers, the same set of variables plus information pertaining to antiretroviral treatment (ART) and HIV-progression were extracted either from the PMTCT registry system or birth records. In the hospital all newborns were weighed immediately after birth using calibrated pediatric scale. The results of the measurements were recorded to the nearest 100 g.

The outcome variable of the study was birth weight dichotomized as normal birth weight (2500–4000 g) or LBW (birth weight less than 2500 g). Other independent variables (potential confounders) considered in the study include maternal age, sex of the newborn, usual place residence (urban, rural), gravidity, premature birth, maternal weight during the third trimester of gestation, anemia, and occurrence of obstetric complications (chronic hypertension, gestational diabetes mellitus, maternal anemia, preeclampsia).

### Data management and analysis

The extracted information was checked for completeness, entered into EPI INFO version 7.0 software and then exported to SPSS version 23 for analysis. Data were summarized using frequency distributions and measures of central tendency and dispersion. Depending on the nature of the variables, basic characteristics of the HIV-positive and -negative mothers were compared using chi-square or independent t-test. The statistical assumptions of the two tests – adequacy of the expected frequency for chi-square test and normality of distribution and homoscedasticity for the latter – were tested following standard procedures. Bivariable and multivariable binary logit models were fitted for examining the association between HIV and birth weight status. In the multivariable model, the association was further adjusted for multiple potential confounders. Potential confounders were identified by running multiple simple logistic regression analyses and variables that had *p*-value of 0.25 or less were considered potential confounders, thus adjusted in the multivariable model. Crude (COR) and adjusted odds ratio (AOR) with 95% confidence intervals (CI) used for summarizing the findings of the analysis. The goodness-of-fit of the model assessed using Hosmer –Lemeshow statistics.

### Ethical considerations

Ethical clearance was secured from the institutional review board (IRB) of Hawassa University, College of Medicine and Health Sciences. To assure privacy, identified data were extracted from the medical records.

## Results

### Socio-demographic characteristics

The data of 529 mother-baby pairs (252 exposed and 277 non-exposed subjects) deemed fit for the analysis. Comparison of the mean (±SD) age of the study participants suggested HIV-positive mothers were significantly older (28.5 ± 4.6 years) than their HIV-negative counterparts (25.9 ± 5.0 years) (*p* <  0.001). Similarly, higher proportions of HIV-positive mothers (72.6%) were urban residents as compared to the HIV-negative women (58.8%) (*p* = 0.001). The HIV-positive women also had more frequent pregnancy experiences than their counterparts (*p* <  0.001). No significant differences were observed between the two groups in terms of sex of the newborn (*p* = 0.240) (Table 1).

**Table 1:** Socio-demographic and obstetric characteristics of HIV-negative and -positive women, HU-SCH, 2018

Characteristics	HIV-negative (***n*** = 277)		HIV-positive (n = 252)		***P***-value
	Freq	%	Freq	%	
Mother’s age at birth (years)					
15–19	21	7.6	7	2.8	< 0.001*
20–24	81	29.2	38	15.1	
25–29	112	40.4	95	37.7	
30–34	38	13.7	84	33.3	
35–39	21	7.6	24	9.5	
40 or above	4	1.4	4	1.6	
Gravidity					
1	102	36.8	29	11.5	< 0.001*
2–3	112	40.4	143	56.7	
4 or more	63	22.7	80	31.7	
Place of residence					
Urban	163	58.8	183	72.6	0.001*
Rural	114	41.2	69	27.4	
Sex of the newborn					
Female	146	52.7	120	47.6	0.240
Male	131	47.3	132	52.4	

### Mode of delivery and obstetric complications

Comparable proportions of HIV- negative (57.0%) and - positive (57.9%) mothers had spontaneous vaginal delivery (*p* = 0.192) and almost similar proportions, 88.8 and 93.3%, respectively, had term pregnancy (37–42 weeks of gestation) (*p* = 0.835). Table-2 compares the magnitudes of selected obstetric and medical complications between the two groups and no statistically meaningful differences were observed in terms of prevalence of premature rupture of membrane (PROM) and chronic hypertension (*p* <  0.05). However, when compared to HIV-negative women, HIV-positives had significantly higher prevalence of urinary tract infection (UTI) and sexually transmitted infection (STI); and lower frequencies of placenta previa, abruption, and preeclampsia (*p* <  0.05).

The mean (± SD) hemoglobin level in HIV-negative (12.5 ± 1.6 g/dl) and -positive (12.6 ± 1.5 g/dl) mothers was more or less the same (*p* = 0.510) and the prevalence of anemia (hemoglobin below 11 g/dl) was balanced (*p* = 0.906). On the other hand, higher proportion of HIV-positive women had third trimester body weight less 50 kgs (*p* <  0.001) (Table 2).

**Table 2:** Distribution of obstetric factors among HIV negative and positive mothers delivered in HUCSH, 2018

Characteristics	HIV-negative (***n*** = 277)		HIV-positive (n = 252)		***P***-value
	Freq	%	Freq	%	
Gestational age (in weeks)					
Pre-term	20	7.2	10	4.0	0.192
Term	246	88.8	235	93.3	
Post-term	11	4.0	7	2.8	
Mode of Delivery					
Simple vaginal delivery	158	57.0	146	57.9	0.835
CS or assisted vaginal delivery	119	43.0	106	42.4	
Premature Rupture of Membrane					
No	240	86.6	221	87.7	0.131
Yes	37	13.4	31	12.3	
Placental abruption					
No	259	93.5	248	98.4	0.005*
Yes	18	6.5	4	1.6	
Placenta previa					
No	265	95.7	250	99.2	0.010*
Yes	12	4.3	2	0.8	
Chronic hypertension					
No	267	96.4	244	96.8	0.783
Yes	10	3.6	8	3.2	
Preeclampsia					
No	235	84.8	243	96.4	< 0.001*
Yes	42	15.2	9	3.6	
STI during pregnancy					
No	272	98.2	226	89.7	< 0.001*
Yes	5	1.8	26	10.3	
UTI during pregnancy					
No	264	95.3	212	84.1	< 0.001*
Yes	13	4.7	40	15.9	
Anemia					
No	227	82.7	209	82.9	0.906
Yes	48	17.3	43	17.1	
Maternal Weight (kg)					
< 50	7	2.5	27	10.7	< 0.001*
> =50	270	97.5	225	89.3	
Malaria during pregnancy					
No	266	96.0	243	96.4	0.810
Yes	11	4.0	9	3.6	

### Treatment-related conditions among HIV-positive subjects

The vast majority (84.5%) of the HIV-positive mothers were on Highly Active Antiretroviral Therapy (HAART) and 89.2% initiated the treatment prior to the current pregnancy. Most of the HIV-positive mothers were on a combination of TDF-3TC-EFV treatment regimen. The mean (±SD) baseline CD_4_ count at diagnosis was 343(± 254) cells/mm^3^ and the corresponding level during the index pregnancy was 556 (± 275) cells/mm^3^. During the pregnancy, 75.1% of the women had CD_4_ count above 350 cells/mm^3^. According to the World Health Organization (WHO) HIV/AIDS staging system, 86.1% were in the Stage-I category (Table 3).

**Table 3:** Treatment related conditions of HIV positive mothers who delivered in HUCSH, 2018

Characteristics	Frequency	Percent
HAART exposure (n = 252)		
No	39	15.5
Yes	213	84.5
HAART initiated (*n* = 213)		
During pregnancy	23	10.8
Before pregnancy	190	89.2
ART Regimen (n = 213)		
TDF-3TC-EFV	135	63.4
AZT-3TC-NVP	53	24.9
TDF-3TC-NVP	25	11.7
Baseline CD4 count (cells/mm^3^) (n = 213)		
< 200	69	32.4
201–350	59	27.7
> 350	85	39.9
CD4 level during the pregnancy (cells/mm^3^) (n = 213)		
< 200	16	7.5
201–350	37	17.4
> 350	160	75.1
WHO clinical staging (n = 252)		
Stage I	217	86.1
Stage II	17	6.7
Stage III	11	4.4
Stage IV	7	2.8

### Gestational age and birth weight of the newborns

**Fig. 2:**
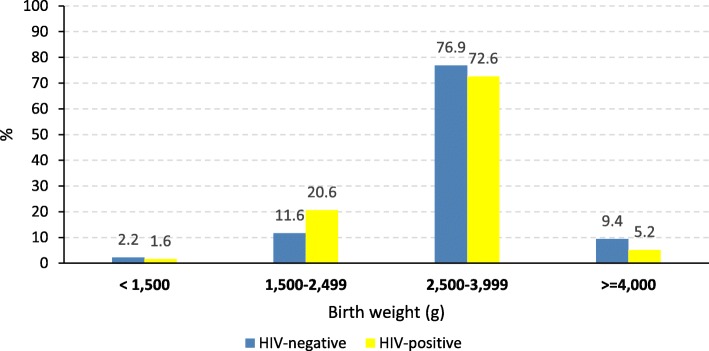
Birth weight distribution of babies born to HIV-negative and -positive mothers at HUCSH, Ethiopia, 2018

The mean (± SD) birth weight was significantly lower in infants born to women infected with HIV (3.0 ± 0.6 kg) than those born to non-infected women (3.1 ± 0.7 kg) (*p* = 0.020). The prevalence of LBW was significantly higher in the exposed group (22.2%) than the non-exposed group (13.7%) (*p* = 0.011); however, no significant difference observed between the groups in the proportions of infants born with very low birth weights (< 1500 g) (Fig. 2).

### Association between maternal HIV status and birth weight

In the bivariable logistic regression analyses; maternal HIV status and other eight covariates (maternal age, place of residence, anemia, chronic hypertension, preeclampsia, gravidity, maternal weight, placental abruption, and STI during pregnancy) had *p*-value less than 0.25, thus considered as candidate variables for the multivariable model. In the multivariable model maternal HIV status and four other variables (place of residence, maternal anemia, chronic hypertension and preeclampsia) sustained their statistical significance.

In the bivariable model HIV-positive women had 1.80 (COR = 1.80, 95% CI: 1.14–2.83) times increased odds of LBW as compared to their counterpart. In the multivariable model adjusted for other potential confounders, HIV-positive mothers demonstrated 4 times increased odds of giving birth to LBW baby (AOR = 4.03, 95% CI: 2.01–8.06). Other significant predictors of LBW were rural place of residence (AOR = 2.04, 95% CI: 1.16–3.60), prenatal anemia (AOR = 3.17, 95% CI: 1.71–5.90), chronic hypertension (AOR = 3.68, 95% CI: 1.10–12.46) and preeclampsia (AOR = 6.80, 95% CI: 3.00–15.38) (Table 4).

**Table 4:** Results of bivariableand multivarible Logistic regression analyses showing determinants of LBW among mothers who delivered at HU-CSH, 2018

Characteristics	Birth Weight		COR(95%CI)	AOR(95%CI)
	LBW	NBW		
HIV Status				
Negative	38	239	1	1
Positive	56	196	1.80(1.14,2.83)	4.03(2.01,8.06)*
Mother’s age at birth				
< 25	23	124	1	1
25–29	33	174	1.00(0.57,1.83)	1.08(0.49,2.40)
30–34	29	93	1.68(0.91,3.10)	1.47(0.62,3.51)
35+	9	44	1.10(0.47,2.56)	1.06(0.35,3.28)
Anemia				
No	64	374	1	1
Yes	30	61	2.90(1.72,4.80)	3.17(1.71,5.90)*
Residence				
Urban	50	296	1	1
Rural	44	139	1.87(1.19,2.95)	2.04(1.16,3.60)*
Chronic hypertension				
No	87	424	1	1
Yes	7	11	3.10(1.17,8.23)	3.68(1.10,12.46)*
Preeclampsia				
No	73	405	1	1
Yes	21	30	3.88(2.12,7.15)	6.80(3.00,15.38)*
Gravidity				
1	17	114	1	1
2–3	46	209	1.48(0.81,2.70)	1.28(0.57,2.87)
4+	31	112	1.86(0.97,3.54)	1.20(0.48,3.04)
Maternal third trimester weight (kg)				
< 50	9	25	1	1
50+	85	410	0.58(0.26,1.28)	0.58(0.23,1.50)
Placental abruption				
No	87	420	1	1
Yes	7	15	2.25(0.89,5.69)	1.70(0.45,6.45)
STI during pregnancy				
No	84	414	1	1
Yes	10	21	2.35(1.07,5.17)	2.51(0.98,6.36)

*p - value < 0.05

## Discussion

The aim of the current study was to examine the association between maternal HIV serostatus and LBW and the analysis suggested that HIV-positive women were four times more likely than HIV-negative mother to give birth to LBW infants. Similarly, infants born to HIV-negative women were heavier on average by 100 g. The other variables which represent independent risk factors for LBW were rural place of residence, maternal anemia, chronic hypertension and preeclampsia.

Previous studies have also availed evidence on the positive association between maternal HIV infection and birthweight. A meta-analysis that pooled the findings of 52 studies concluded that maternal HIV infection was significantly related to LBW (OR = 1.7) and preterm delivery (OR = 1.6) [17]. Similarly, another systematic review reported HIV infection increased risk of low birthweight (RR = 1·6) and preterm birth (relative risk (RR) =1·50) especially in the sub-Saharan Africa region [28]. Regarding studies conducted in Ethiopia, two hospital-based studies conducted in Northwestern [21] and Northern [22] part of the country reported stronger associations (OR of 5.2 and 6.1, respectively).Contrary to our results, a study in Southwestern Ethiopia came across with no significant association but the study might have been under powered as it enrolled smaller number of HIV positive subjects [29]. Significant relationships had also been reported in studies conducted in other developing countries including Malawi [18], Kenya [19] and Cameroon [30].

Maternal HIV-infection may lead to low birthweight through multiple and complex biological and psychosocial pathways. A systematic review postulated that HIV-induced damage to human immune system may mediate HIV-LBW relationship [17]. HIV infection may also raise susceptibility to LBW through predisposing to obstetric complications including anemia that can independently restrict birthweight [31, 32]. A recent study has suggested that protease inhibitor-based antiretroviral regimens may lead to LBW and prematurity [33]. Furthermore, under nutrition including micronutrient deficiencies which are more frequently observed in HIV-infected pregnant women may predispose to LBW. HIV infection reduces appetite, causes malabsorption of nutrients, alters metabolism and increases the demand for essential nutrients to cause wasting syndrome and thus LBW [34]. Previous cross-sectional studies that compared the micronutrient status of HIV-infected and non-infected women have indicated that vitamin A, iron, folic acid and zinc deficiencies are more frequent in HIV positive women [35–38]. In addition, HIV positive and -negative women may not have comparable socioeconomic backgrounds and the latter are more likely to be exposed to poverty, lack of social support, stress and substance use, which all can lead to LBW.

We also identified maternal anemia as an independent predictor of LBW with an adjusted odds ratio of 3. A metanalysis of 68 cohort and case-control studies also recognized anemia as a risk factor for LBW with OR of 1.2 [31]. Maternal anemia may limit oxygen delivery to fetus to cause growth restriction [39]. Low maternal hemoglobin may also alter placental mass, size, morphology and vascularization to attenuate fetal growth [40–42]. Anemia may also indirectly designate sub-optimal maternal nutrition which is an important risk factor of LBW [40].

In this study, the odds of giving LBW births were higher among mothers who had chronic hypertension (OR = 3.7) or preeclampsia (OR = 6.8). A systematic review concluded pregnant women with chronic hypertension had 17 to 28% high incidences of LBW and preterm delivery, respectively [43]. A study in Norway concluded, preeclampsia reduced birth weight by 5 to 25% depending on the severity and time of onset of the disorder [44]. It has been hypothesized that maternal hypertensive disorders attenuate birth weight through multiple mechanisms including decreasing uteroplacental blood flow and causing placental insufficiency [45].

The findings of the study should be interpreted in consideration of the following limitations. First, as the study was limited to a hospital, the external validity of the study can somehow be questioned. Second, HIV-positive and -negative women may not have similar background characteristics in pertinent variables that can independently predict LBW, thus the findings might have been compromised by selection bias. Third, as the study was carried out in a tertiary hospital, the prevalence of LBW can be overestimated in both groups, especially in the HIV-negative group. Here it is important to note that HIV-negative women would end up in giving birth in advanced facilities probably due to the occurrence of obstetric complications that require specialized care. Yet this may not be necessarily the case for the HIV-positive women because they would normally be required to give birth in higher facilities. Fourth, missingness of key variables (e.g. socio-economic status, substance use, etc.) that emanates from the use of secondary data could have resulted in residual confounding. Fifth, as most of the HIV-positive women were on ART, the natural relationship between the variables of interest could have been underestimated. And sixth, as we measured the association between the exposure and the outcome using odds ratio, the true relationship might have been overestimated. There is a universal agreement that with increasing prevalence of the outcome, OR tends to overestimated the relative risk.

## Conclusion

Our finding suggests that maternal HIV infection is associated with increased risks of low birthweight. Other independent risk factors of low birth weight were rural place of residence, maternal anemia, chronic hypertension and preeclampsia. HIV prevention activities are likely to help for reducing the incidence of LBW.

## Abbreviations

AOR: Adjusted odds ratio
CI: Confidence interval
COR: Crude odds ratio
DHS: Demographic and health survey
HAART: Highly Active Anti Retroviral Therapy
HIV: Human Immunodeficiency Virus
IRB: Institutional review board
LBW: Low birth weight
PROM: Premature Rupture of Membrane
SD: Standard Deviation
SPSS: Software package for social sciences
STI: Sexually Transmitted Infections.
